# *In vitro* investigations of ABC transporters of the human liver – advantages and surprises

**DOI:** 10.1186/2047-783X-19-S1-S18

**Published:** 2014-06-19

**Authors:** Lutz Schmitt

**Affiliations:** 1 Institute of Biochemistry, Heinrich Heine University,40225 Düsseldorf, Germany

## 

The hepatocyte contains a battery of membrane transporters in the sinusoidal and canalicular membrane that guarantee the regulated import and export of nutrients, messengers and toxic compounds. In the canalicular membrane, a number of important ABC (ATP binding cassette) transporters are located. Three of these primary active transporters, BSEP (bile salt export pump, ABC B11), MDR3 (multidrug resistance protein 3, ABC B4) and ABC G5/G8 catalyze the secretion of bile acids (BSEP), phosphatidylcholine (MDR3) and cholesterol (ABC G5/G8). These three molecules form mixed micelles in bile and thereby reduce the harmful action of unbound bile salts on membranes.

BSEP, which was initially named s-Pgp (sister of P-gp) and MDR3 share a high sequence identity with MDR1 (P-glycoprotein, ABC B1), which is the hallmark of the family of multidrug resistance ABC transporter present in every eukaryotic cell. For BSEP the identity is approximately 70% and for MDR3 even 85%, respectively. Nevertheless, MDR3 is specific for phosphatidylcholine lipids and BSEP is specific for bile acids, while MDR1 is capable of translocating a myriad of structurally unrelated compound across biological membranes. This of course raises the question, which residues impose specificity in the case of BSEP and MDR3 and which residues impose promiscuity in the case of MDR1.

Homology models of BSEP and MDR3 were generated based on the crystal structures of Sav1866 and mouse P-gp to obtain initial information about the putative three-dimensional location of certain amino acid residues of BSEP and MDR3. However, one has to stress that these “structures” represent only models that have to be used with proper caution. The homology model of MDR3 was helpful in providing a structural explanation for the non-functionality of the MDR3 mutant H1231Y. The mutant protein was properly located to the plasma membrane, but displayed a severe substrate transport phenotype. Histidine 1231 corresponds to the histidine of the H-loop, a residue that is critical for ATP hydrolysis in many other ABC transporter systems. Thus, exchange of His against Tyr in position 1231 of MDR3 will impair ATP hydrolysis in one of the two ATP binding site drastically reducing transport activity of MDR3 H1231Y [1].

To study the transport cycle and the importance of mutations within these transporters on their function, overexpression systems are required. We decided to use the methylotrophic yeast *Pichia pastoris* for the overexpression of two of the three transporters, BSEP and MDR3, respectively. Initially we encountered severe problems in cloning the transporters genes in *E. coli* employing standard approaches. Only after establishing a cloning strategy that is entirely based on *Saccharomyces cerevisiae*, both genes could be successfully cloned [2]. This allowed us to combine the speed and ease of cloning of this yeast system for the wild type genes and mutations that were identified in patients.

Having this tool in hand, we overexpressed BSEP in yeast to study its transport function in plasma membrane vesicles. This allowed us to establish structure-function relations for a new clinically relevant mutation (G374S) that resulted in a phenotype between PFIC-2 and BRIC-2. Although membrane targeting was normal, e.g. targeting to the canalicular membrane was evident, transport activity of the G374S was strongly impaired [3]. The Sav1866-based homology model of BSEP suggested that Gly 374 is lining the translocation pore of BSEP. Thus, an exchange to serine would impose steric restriction within the pore, thereby influencing the substrate spectrum as evident from the bile acid transport assays.

Based on the expression levels of BSEP and MDR3, purification protocols for both membrane proteins were successfully established [4]. Thus, next to the transport assay that is based only highly enriched yeast plasma membranes, purified protein is now also available in the detergent-solubilized state. While the transport assay can be used for BSEP to analyze a possible influence of a mutation on the transporter activity of the protein, purified MDR3 can be used to determine the influence of a mutation on ATPase activity and / or stimulation or inhibition of this activity.

In summary, the established yeast overexpression system combines the ease and speed of yeast cloning approaches with the possibility to overexpress and purify both proteins in a quantity and homogeneity sufficient for biochemical and biophysical investigations of the function of these two ABC transporters. Furthermore, the speed with which mutants can be obtained will allow an in depth characterization of clinically relevant mutations of both transporters with respect to function and spectrum of transported substrates.
